# AP-1 confers resistance to anti-cancer therapy by activating XIAP

**DOI:** 10.18632/oncotarget.23897

**Published:** 2018-01-03

**Authors:** Yuan Wang, Guo-Hui Wan, Ying-Min Wu, Hong-Sheng Wang, Hai-Fang Wang, Ge Zhang, Lin-Lin Lu, Zi-Qian Li, Ka-Ying Chan, Yan Zhou, Shao-Hui Cai, Yi-Fei Qi, Jun Du

**Affiliations:** ^1^ Department of Microbial and Biochemical Pharmacy, School of Pharmaceutical Sciences, Sun Yat-sen University, Guangzhou 510006, China; ^2^ Department of Pharmacology, School of Pharmaceutical Sciences, Jinan University, Guangzhou 510632, China

**Keywords:** drug resistant, HDAC inhibitor, JNJ26481585, AP-1, XIAP

## Abstract

The underlying cause of treatment failure in many cancer patients is intrinsic and acquired resistance to chemotherapy. Recently, histone deacetylase (HDAC) inhibitors have developed into a promising cancer treatment. However, resistance mechanism induced by HDAC inhibitors remains largely unknown. Here we report that a HDAC inhibitor, JNJ-2648158 induced transcription of XIAP by activating AP-1 expression, which conferring resistance to chemotherapeutics. Our results showed that high expression of c-Fos caused by HDAC inhibitor promoted AP-1 formation during acquired resistance towards chemo-drugs, indicating an extremely poor clinical outcome in breast cancers and liver cancers. Our study reveals a novel regulatory mechanism towards chemo-drug resistance, and suggests that XIAP may serve as a potential therapeutic target in those chemo-resistant cancer cells.

## INTRODUCTION

Developing drug-resistant tumors in patients is a major obstacle in both conventional chemotherapeutics and novel targeted therapeutics [1]. About 90% patients obtain chemotherapeutic failure due to generation of drug-resistant cancer cells [2], even in their initial treatment [3]. The major mechanisms of drug-resistance in cancer cells are diversified and complicated processes, including activating of DNA repair, decreasing drug influx, confiscating of drugs within intracellular organelles, increasing drug efflux, disabling of apoptosis pathways, and triggering of immune response etc. [4, 5]. Recently, novel mechanism involved in drug resistant cancer cells has been largely focused on the high expression of ATP-binding cassette (ABC) transporter proteins. They showed that the overexpressed ABC transporters through efflux the anticancer drugs from the cytoplasm of tumor cells to reduce drugs accumulation in cells and then cause the very low therapeutic result [6, 7].

Histone deacetylase (HDAC) inhibitors have been approved for clinical treatment of various cancers including cutaneous T cell lymphoma [8], breast [9], non-Hodgkin's lymphoma and mantle cell lymphoma [10, 11]. Previous study has developed a novel second-generation of HDAC inhibitor JNJ-2648158 (Quisinostat, hereafter referred to JNJ for short) and found that JNJ exerted potent antitumor activity against rhabdomyosarcoma (RMS) *in vitro* and *in vivo* by engaging mitochondrial apoptosis [12]. Recently, other groups have shown that JNJ was able to prolong pharmacodynamic response and translate into complete tumor growth inhibition in Ras mutant colon carcinoma xenografts, thus repressing growth of colorectal liver metastases [13]. Furthermore, JNJ was found to up-regulate expression of p21 and result in G1 phase arrest by increasing p53 acetylation at K382/K373 sites [14]. From the above attempts, JNJ may function as a promising adjunct treatment, and thus we examined its potential effects towards chemo-resistant cancer patients.

In this study, we used Adriamycin (ADR)-resistant breast cancer cells MCF/ADR to examine the effects of JNJ treatments. Interestingly, we identified an extremely poor outcome in MCF7/ADR cells with JNJ treatment, and showed that JNJ treatment promoted more resistance toward ADR instead of sensitizing the cells to this chemo drug. We further studied the underlying molecular mechanism that contributes to the resistant effects caused by JNJ treatment. A novel activating signaling pathway was subsequently identified in our study.

## RESULTS

### JNJ promotes ADR resistant effects in MCF7/ADR cells

**Figure 1:**
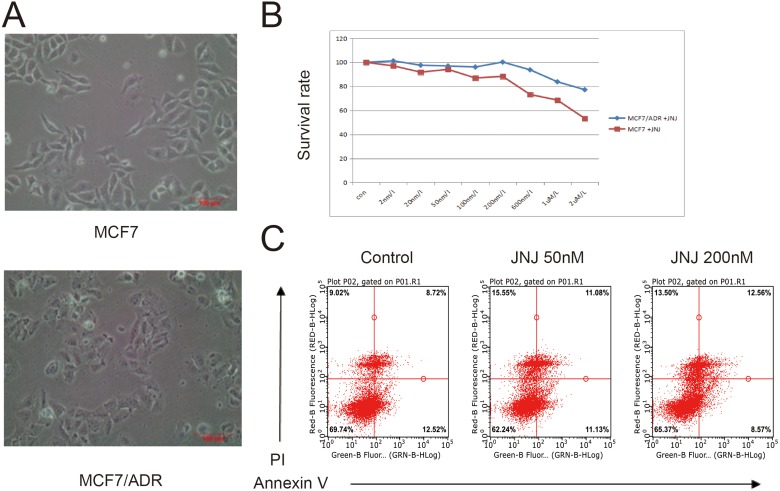
JNJ promotes ADR resistant effects in MCF7/ARD cells (**A**) Characterization of MCF7 and MCF7/ADR cells. (**B**) JNJ induces resistant effects in MCF7/ARD cells by comparing with MCF7 cells. Different concentrations of JNJ were incubated for 24 h and survival rates were measured by MTT assay. (**C**) Treatment of JNJ showed little apoptosis effects in MCF7/ARD cells. Cells treated with or without JNJ were stained with PI and Annexin V, and apoptotic cells were analyzed by Flow Cytometry.

JNJ specifically targets against HDAC1, HDAC2, HDAC4, HDAC10 and HDAC11 with IC_50_ of 0.11 nM, 0.33 nM, 0.64 nM, 0.46 nM, and 0.37 nM, respectively. In order to completely suppress most HDACs in the certain cells such as MCF7 and MCF7/ARD cells (Figure 1A), we applied a maximal concentration of 50 nM JNJ for the treatment and we found that the toxicity of 50 nM JNJ was mild in the cell (Figure 1B). Treatment of 50 nM JNJ also had little effect on cells apoptosis rate (Figure 1C). By treating with JNJ, the therapeutic effect towards MCF7/ADR and MCF7 cells were investigated by MTT assay (Table 1). The IC_50_ values of ADR on MCF7 and MCF7/ADR cells after pre-treated with JNJ for 48 h was 58.16 μM and 293.53 μM, respectively. The drug resistance (DR) factor of MCF7/ADR cells was 5.05-fold higher than the naïve MCF7 cells (Table 1). Interestingly, two opposite results were observed by apply treatment of JNJ. Naïve MCF7 cells were sensitive to ADR with pretreated with JNJ (IC_50_ reduced 36.81%), however MCF7/ADR cells showed more resistant effects toward ADR with pretreated with JNJ (IC_50_ induced 31.36%). JNJ is able to promote ADR resistant effects in MCF7/ADR cells.

**Table 1:** Determination of IC_50_ in MCF7 and MCF7/ADR cells

Anticancer drugs/IC_50_	MCF7 μM	MCF7/ADR μM	Resistant fold
Adriamycin	58.16	293.53	5.05
Adriamycin and JNJ (50 nM)	36.75	385.58	10.49
IC_50_ Promotion rate	−36.81%	31.36%	

ADR, Adriamycin. Concentration gradient of ADR for MTT is 0.03448, 0.3448, 3.448, 34.48, 103.448, 344.8, 689.65, 1379.31 μM IC_50_ Promotion rate % = (Adriamycin + JNJ(50nM)) group IC_50_value – Adriamycin group IC_50_value)*100%/Adriamycin group IC_50_value. Resistant fold = MCF7/ADR cells IC_50_value/ MCF7 cells IC_50_value.

### JNJ induces expression of XIAP in MCF7/ADR cells

**Figure 2:**
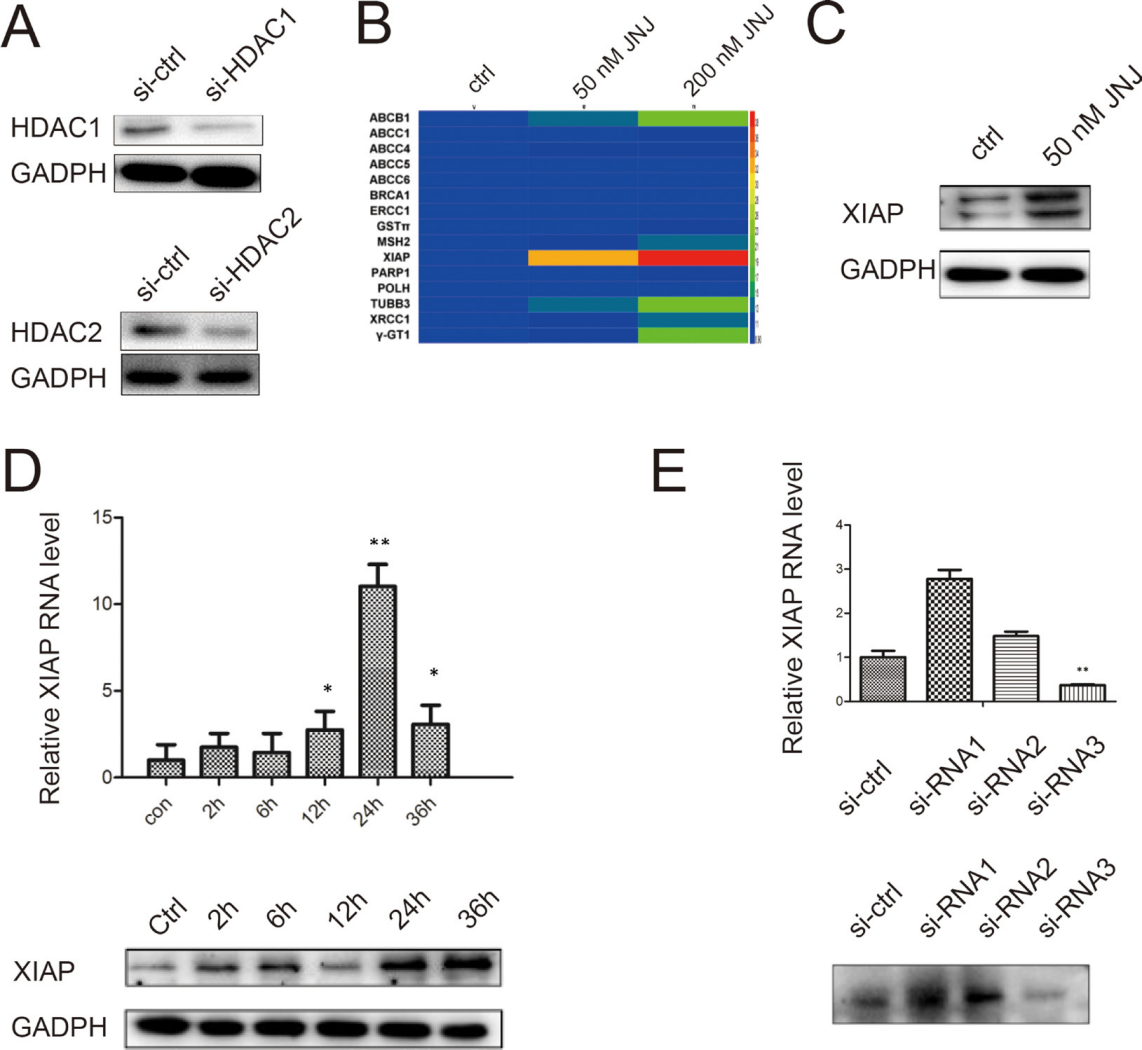
JNJ induces XIAP expression in MCF7/ARD cells (**A**) HDAC1 and HDAC2 were knockdowned by siRNAs and there protein expression levels were validated by western blot. (**B**) Expression of resistant genes was analyzed in MCF7/ARD cells after treated with JNJ. Red or green color in the heat map means an increased or a decreased of mRNA level. (**C**) XIAP was induced after treated with JNJ for 24 h in MCF7/ARD cells. (**D**) XIAP is induced in both RNA level and protein level after treated with JNJ and its induction was time dependent. The RNA level of XIAP was measured by quantitative real time PCR assay, while the protein level of XIAP was measured by western blot. (**E**) XIAP was knockdowned by siRNAs. The knockdown effects were measured both in the RNA level and protein level.

To investigate how JNJ induces elevated resistance in MCF7/ADR cells, we first examined whether the resistant effect was caused by inhibiting activities of HDACs. Knockdown of HDAC1 and HDAC2 by siRNAs were confirmed by western blot (Figure 2A). Cell viability in MCF7/ADR cells with knockdown HDAC1 or HDAC2 was assessed by MTT to evaluate the IC_50_ change of Adriamycin+ JNJ (50 nM). The IC_50_ of MCF7/ADR was upregulated by 21.79% in HDAC1 knockdown group and 35.16% in HDAC2 knockdown group, respectively (Table 2 and 3). It indicates that knockdown HDACs did not help to decrease the resistant effect generated by JNJ, and thus other signal pathways may contribute to the resistance by JNJ in MCF7/ADR cells. To identify the potential target genes for the resistance, we applied the screening of several known drug resistance factors and apoptosis inhibitory factors by measuring their changes in RNA levels. After treated with JNJ for 24 h, we identified that XIAP was significantly upregulated (Figure 2B). Further, induction of XIAP level was validated by western blot (Figure 2C). To analyze the temporal expression changes of XIAP, we measured XIAP expression level in both RNA level and protein level in different time points pretreated the cells with JNJ. XIAP was upregulated in time dependent manner and reached the maximal induction at 24 h (Figure 2D). To examine whether XIAP contributes to the resistant effect in MCF7/ADR cells treated with JNJ, we knockdown XIAP by siRNAs (Figure 2E) and measured cell viability by MTT assay (Table 4). Knockdown XIAP was able to reduce resistance caused by JNJ toward ADR treatment (0.89 fold) in MCF7/ARD cells (Table 4). XIAP may serve as the potential therapeutic target for the resistance generated by JNJ.

**Table 2:** Determination of IC_50_ in MCF7 /ADR cells with siHDAC1

Anticancer drugs/IC_50_	MCF7/ADR cells transfected with siNC	MCF7/ADR cells transfected with siHDAC1
Adriamycin group	234.25	251.58
Adriamycin+ JNJ (50 nM) group	317.58	306.39
IC_50_ Promotion rate	35.57%	21.79%

ADR, Adriamycin. Concentration gradient of ADR for MTT is 0.03448, 0.3448, 3.448, 34.48, 103.448, 344.8, 689.65, 1379.31 μM. IC_50_ Promotion rate% = [(Adriamycin + JNJ(50 nM)) group value – Adriamycin group value]*100%/Adriamycin group value.

**Table 3:** Determination of IC_50_ in MCF7 /ADR cells with siHDAC2

Anticancer drugs/IC_50_	MCF7/ADR cells transfected with siNC	MCF7/ADR cells transfected with siHDAC2
Adriamycin group	235.18	287.41
Adriamycin+ JNJ (50 nM) group	291.68	388.47
IC_50_ Promotion rate	24.0%	35.16%

ADR, Adriamycin. Concentration gradient of ADR for MTT is 0.03448, 0.3448, 3.448, 34.48, 103.448, 344.8, 689.65, 1379.31 μM. IC_50_ Promotion rate% = [(Adriamycin + JNJ (50 nM)) group value – Adriamycin group value]*100%/Adriamycin group value.

**Table 4:** Determination of IC_50_ in MCF7 /ADR cells with siXIAP

Anticancer drugs/IC_50_	MCF7/ADR cells transfected with siNC	MCF7/ADR cells transfected with siXIAP	Resistant fold
Adriamycin	223.18	225.95	1.012
Adriamycin + JNJ (50 nM)	262.25	233.06	0.89
IC_50_Promotion rate	17.5%	3.1%	

ADR, Adriamycin. Concentration gradient of ADR for MTT is 0.03448, 0.3448, 3.448, 34.48, 103.448, 344.8, 689.65, 1379.31 μM. IC_50_Promotion rate % = (Adriamycin + JNJ (50 nM)) group IC_50_ value – Adriamycin group IC_50_ value)*100%/Adriamycin group IC_50_value. Resistant fold = MCF7/ADR cells transfected with siXIAPIC_50_value/ MCF7/ADR cells transfected with siNCIC_50_value.

### AP-1 mediated XIAP expression by JNJ

**Figure 3:**
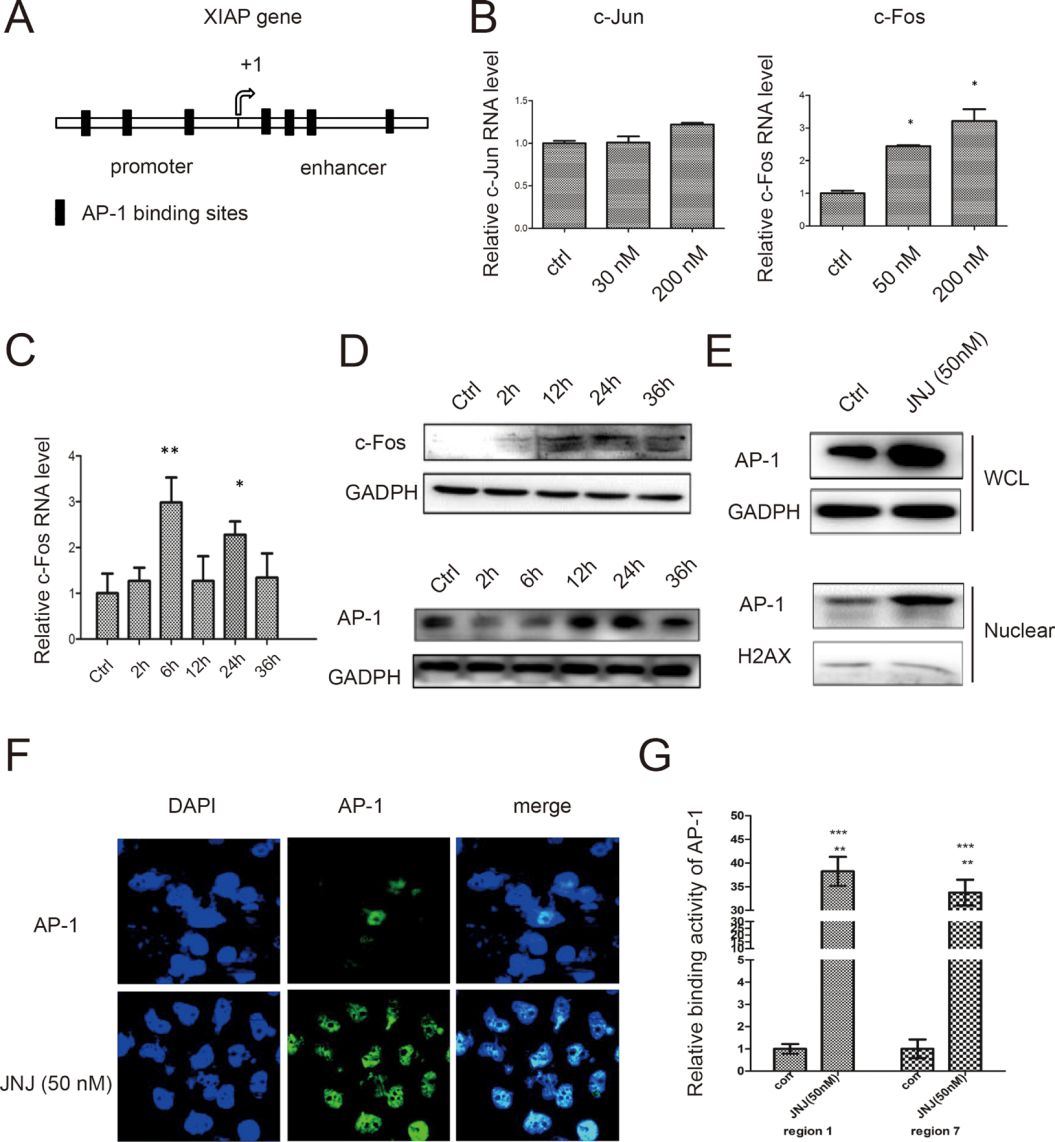
AP-1 mediates XIAP expression by JNJ in MCF7/ARD cells (**A**) Schematic figure of XIAP gene promoter. AP-1 bindings sites are shown as black blocks in both promoter region and enhancer region. (**B**) The RNA expression levels of c-Jun and c-Fos were measured by quantitative real time PCR in MCF7/ARD cells after JNJ treatment. (**C**) The temporal RNA expression of c-Fos was measured in MCF7/ARD cells after JNJ treatment. (**D**) The temporal protein expression levels of c-Fos and AP-1 were measured by western blot in MCF7/ARD cells after JNJ treatment. (**E**) The expression of AP-1 in the whole cell lysate and nuclear fraction was measured by western blot in MCF7/ARD cells after JNJ treatment. (**F**) Localization and expression of AP-1 was measured by fluorescent immunohistochemistry assay in MCF7/ARD cells after JNJ treatment. (**G**) Relative binding activity of AP-1 to XIAP promoter regions was measured by CHIP assay in MCF7/ARD cells after JNJ treatment.

**Figure 4:**
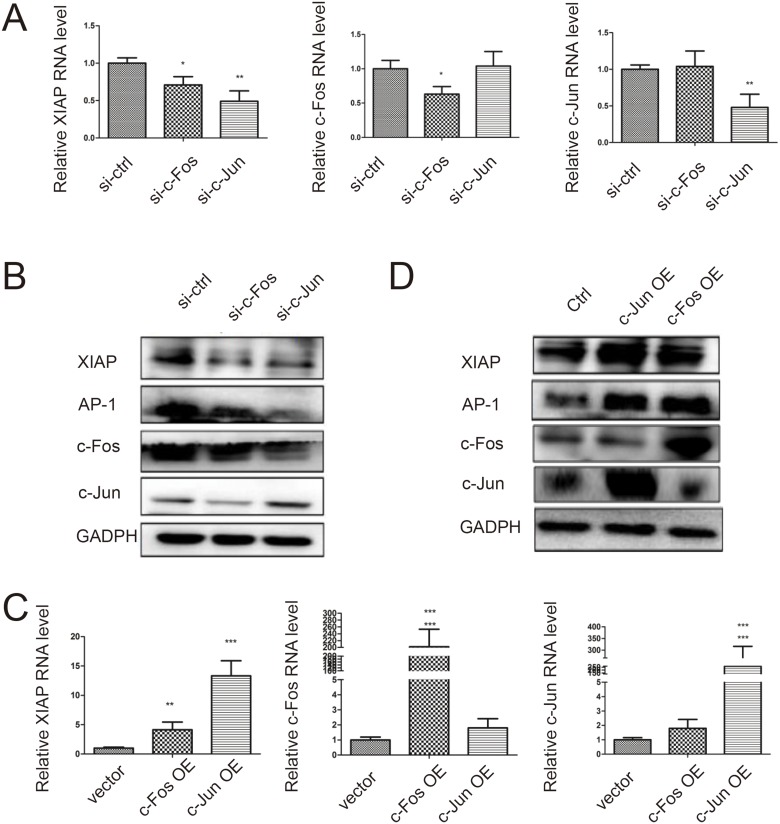
Activating AP-1 formation induces XIAP expression (**A**) The RNA expression level of XIAP is downregulated by inhibiting AP-1 formation. The RNA expression level of XIAP was measured by quantitative real time PCR. The expression of c-Fos and c-Jun was knockdowned by siRNAs and their RNA expression levels were validated by quantitative real time PCR. (**B**) The protein expression level of XIAP is downregulated by inhibiting AP-1 formation. The protein expression level of XIAP was measured by western blot. (**C**) The RNA expression level of XIAP is upregulated by activating AP-1 formation. The RNA expression level of XIAP was measured by quantitative real time PCR. The over-expression of c-Fos and c-Jun were validated by quantitative real time PCR. (**D**) The protein expression level of XIAP is upregulated by activating AP-1 formation. The protein expression level of XIAP was measured by western blot.

To investigate how XIAP was induced by JNJ for the resistant effect, we applied promoter analysis in XIAP gene for transcriptional regulation. We identified seven AP-1 binding sites in both promoter region and enhancer region of XIAP gene (Figure 3A). AP-1 complex is formed by c-Jun and c-Fos, therefore, we further measured the expression level of c-Jun and c-Fos in MCF7/ADR cells treated with JNJ. We found c-Fos was significantly upregulated in RNA level after treated with JNJ while c-Jun remains unchanged (Figure 3B). We further analyzed the temporal expression of c-Fos, and found that c-Fos was upregulated in time dependent manner both in RNA level and protein level (Figure 3C and 3D). Accordingly, AP-1 expression level was found to be consistently upregulated with c-Fos expression (Figure 3D). To further study regulation of AP-1 in JNJ treatment, we examined translocation change of cytosolic AP-1 into the nucleus. We found that AP-1 was significantly translocated in the nucleus after JNJ treatment (Figure 3E). Further, we applied immunofluorescence technique to confirm the increased translocation of AP-1 in the nucleus after treatment of JNJ (Figure 3F). Whether AP-1 directly regulates XIAP in JNJ treatment, we used CHIP assay to analyze the binding activity of AP-1 in XIAP gene promoter and enhancer regions. AP-1 was found to significantly bind to XIAP promoter and enhancer regions after JNJ treatment, indicating that JNJ promotes AP-1 recruitment in XIAP gene and activates the transcription of XIAP gene (Figure 3G). Furthermore, we changed both c-Fos and c-Jun expression level to study regulation of XIAP expression by AP-1 formation. We found that knockdown c-Fos and c-Jun significantly reduced XIAP expression in both RNA level and protein level (Figure 4A and 4B). Knockdown c-Fos and c-Jun by siRNAs was confirmed by qPCR and western blot (Figure 4A and 4B). Moreover, we overexpressed c-Fos and c-Jun by transfected their expressing vectors in the cells, and found that overexpressed c-Fos and c-Jun were able to significantly upregulated XIAP expression in both RNA and protein level (Figure 4C and 4D). Overexpression of c-Fos and c-Jun was validated by qPCR and western blot as well (Figure 4C and 4D). These results support that activating AP-1 formation induces XIAP expression in MCF7/ADR cells treated with JNJ.

### JNJ sensitizes naïve MCF7 cells by downregulating expression of XIAP

**Figure 5:**
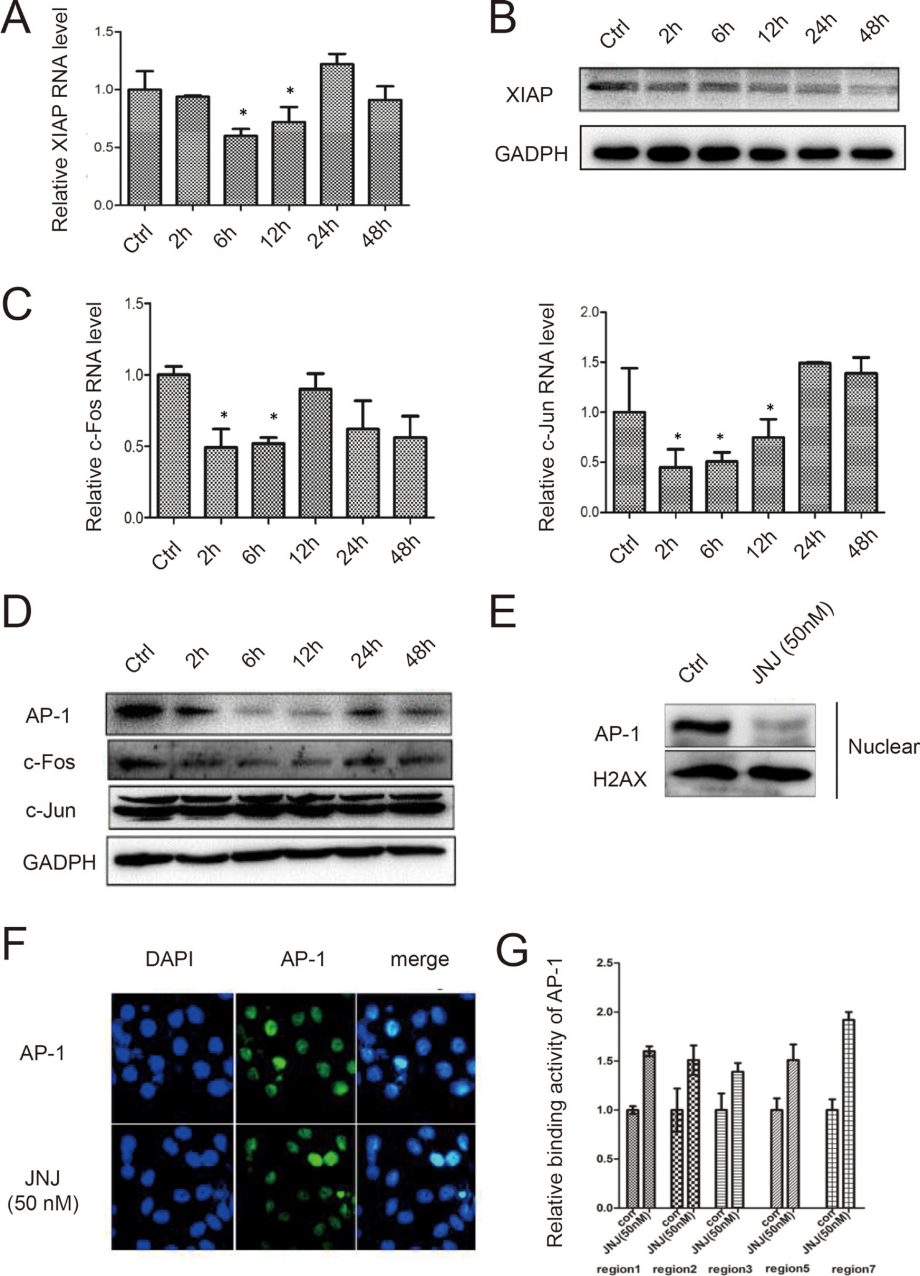
JNJ sensitizes naïve MCF7 cells by inhibiting AP-1 signal pathway and downregulated expression of XIAP (**A**) The temporal RNA expression of XIAP was measured by quantitative real time PCR in naïve MCF7 cells after JNJ treatment. (**B**) The temporal protein expression of XIAP was measured by western blot in naïve MCF7 cells after JNJ treatment. (**C**) The temporal RNA expression of c-Fos and c-Jun were measured by quantitative real time PCR in naïve MCF7 cells after JNJ treatment. (**D**) The temporal protein expression of c-Fos and c-Jun were measured by western blot in naïve MCF7 cells after JNJ treatment. (**E**) AP-1 expression was downregulated in nuclear in naïve MCF7 cells after JNJ treatment. (**F**) Localization and expression of AP-1 was measured by fluorescent immunohistochemistry assay in naïve MCF7 cells after JNJ treatment. (**G**) Relative binding activity of AP-1 to XIAP promoter regions was measured by CHIP assay in naïve MCF7 cells after JNJ treatment.

From Table 1, we observed that treatment with JNJ was able to reduce IC50 of ADR towards to naïve MCF7 cells. To find out whether this sensitivity effect by JNJ is also mediated by the c-Fos/AP-1/XIAP signaling way found in MCF7/ADR cells, we measured the temporal XIAP expression in both RNA level and protein level in MCF7 cells treated with JNJ. XIAP was significantly reduced in time dependent manner (Figure 5A and 5B). In addition, we measured c-Fos and c-Jun expression in naïve MCF7 cells and found that treatment with JNJ decreased c-Fos expression in both RNA and protein level (Figure 5C and 5D). The RNA level of c-Jun was found to be suppressed after JNJ treatment (Figure 5C) but its protein level remained the same (Figure 5D), indicating reduced formation of AP-1 after JNJ may mainly due to the decrease of c-Fos instead of c-Jun. By analysis AP-1 expression in nuclear faction, we found AP-1 significantly reduced in the nucleus after JNJ treatment in naïve MCF7 cells (Figure 5E). Further, we observed mild decrease in AP-1 expression by using immunofluorescence staining (Figure 5F). In addition, CHIP assay also validated the binding activity of AP-1 in XIAP in MCF7 cells (Figure 5G), it suggests that JNJ inhibits AP-1 signaling pathway in naïve MCF7 cells, leading the reduced expression of XIAP, thus promoting sensitivity towards ADR treatment in naïve MCF7 cells.

### ADR resistant effects caused by JNJ occur in other cancers

**Figure 6:**
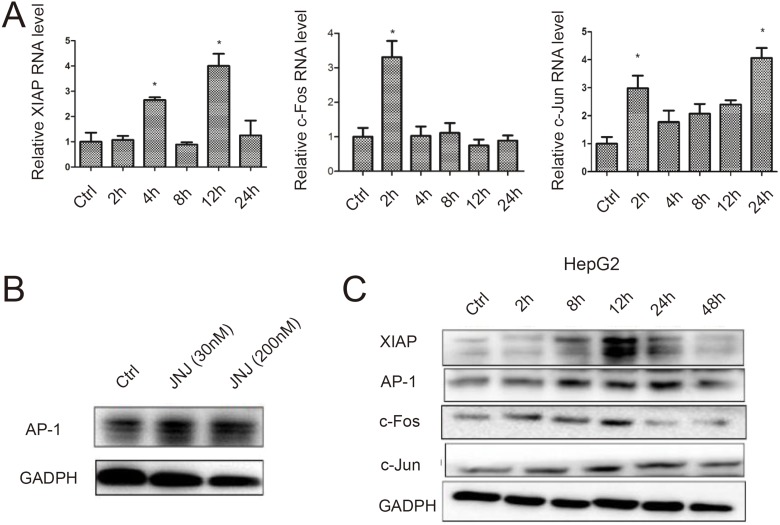
ADR resistant effects caused by JNJ occur in other cancers (**A**) The temporal RNA expression levels of XIAP, c-Fos and c-Jun were measured by quantitative real time PCR in HepG2 cells after JNJ treatment. (**B**) The expression of AP-1 was measured by western blot in HepG2 cells after JNJ treatment. (**C**) The temporal protein expression of XIAP, AP-1, c-Fos and c-Jun were measured by western blot in HepG2 cells after JNJ treatment.

To study universality and importance of c-Fos/AP-1/XIAP signaling pathway in therapeutic resistance, we tested our finding in another cancer such as liver cancer HepG2 cells. By using MTT assay, we found that the IC50 values of ADR on HepG_2_ cells was 20.77 μM, after treated under different ADR concentration gradient for 48 h (Table 5). Consistent with our previous data, after added JNJ, the IC50 of HepG2 to ADR was obviously increased, which is similar to the resistant cells in breast cancer. Further, we analyzed XIAP, c-Fos and c-Jun expression in HepG2 cells treated with JNJ, and found that XIAP, c-Fos and c-Jun were induced in both RNA level and protein level in time dependent manner in HepG2 cells after JNJ treatment (Figure 6A and 6C). Accordingly, AP-1 was found to induce in HepG2 after JNJ treatment (Figure 6B), which is consistent with MCF7/ADR cells. Since XIAP may serve as a potential therapeutic target for the resistance mechanism, we knockdown XIAP in HepG2 cells by siRNAs, and measured cell viability in those knockdown groups by MTT assay (Table 6). Knockdown XIAP in HepG2 was able to significantly reduce resistant effect in liver cancer cells by decreasing the resistant fold to 0.254 with JNJ treatment (Table 6). It further confirmed that XIAP plays a very important role in JNJ-induced drug resistance.

**Table 5:** Determination of IC_50_ in HepG2 cells

Anticancer drugs/IC_50_	Hepg_2_ μM
Adriamycin	20.77
Adriamycin and JNJ (50 nM)	40.97
IC_50_ Promotion rate	97.26%

ADR, Adriamycin. Concentration gradient of ADR for MTT is 0.03448, 0.3448, 3.448, 34.48, 103.448, 344.8, 689.65, 1379.31 μM. IC_50_Promotion rate % = (Adriamycin + JNJ (50 nM)) group IC_50_value – Adriamycin group IC_50_ value)*100%/Adriamycin group IC_50_value. Resistant fold = MCF7/ADR cells IC_50_value/ MCF7 cells IC_50_value.

**Table 6:** Determination of IC_50_ in HepG2 cells with siXIAP

Anticancer drugs/IC_50_	Hepg_2_ cells transfected with siNC	Hepg_2_ cells transfected with siXIAP	Resistant fold
Adriamycin	45.78	17.24	0.377
Adriamycin+ JNJ (50 nM)	76.83	19.55	0.254
IC_50_Promotion rate	67.82%	13.40%	

ADR, Adriamycin. Concentration gradient of ADR for MTT is 0.03448, 0.3448, 3.448, 34.48, 103.448, 344.8, 689.65, 1379.31 μM. IC_50_Promotion rate % = (Adriamycin + JNJ (50 nM)) group IC_50_ value – Adriamycin group IC_50_value)*100%/Adriamycin group IC_50_value. Resistant fold = MCF7/ADR cells transfected with siXIAPIC_50_value/ MCF7/ADR cells transfected with siNCIC_50_value.

## DISCUSSION

Development of drug resistance has always been a tough challenge for cancer treatment due to the aggressive biology. HDACi has been found to induce cell cycle arrest, senescence, apoptosis, and differentiation and many other cellular effects in cancer cells [15]. It has been reported that one of histone deacetylases, vorinostat, can through upregulate p21 lead to arresting the cell cycle in G1 [16]. JNJ was previously reported to upregulate expression of p21, and resulted in G1 phase arrest through increasing p53 acetylation [14], and resulted in apoptosis by engaging the mitochondrial pathway [17]. In our study, by using MTT we compared the naïve tumor strain MCF7, and the resistant strain MCF7/ADR, in response to JNJ treatment to see whether JNJ treatment may serve as an effective adjunct therapeutic in breast cancer. Surprisingly, we found that JNJ can promote apoptosis in naïve MCF7 cells but on the other hand induce more drug resistance instead of sensitivity in MCF7/ADR cells. Furthermore, we revealed the underlying resistant mechanisms caused by JNJ in MCF7/ADR cells, and it is due to the activation of the c-Fos/AP-1/XIAP signaling pathway in MCF7/ADR cells. To apply our finding in a universal manner, we tested cell viability with JNJ treatment in liver cell line HepG2, and confirm that the c-Fos/AP-1/XIAP signaling pathway contributes the resistant effect caused by JNJ. Thus, XIAP may serve as a potential therapeutic target in those resistant cancer cells.

Adriamycin (ADR, a chemotherapeutic drug) is issued as principal medicine for the treatment of solid tumors [18]. But most patients generate subsequent tumor relapse and cause the failure in therapeutic treatment towards ADR in cancer [19–21]. A broad range of cell lines that resist against ADR has been developed and results in the loss of therapeutic efficacy [22, 23]. In our study, ADR was choose to culture the drug resistant MCF7 cell sub clones by developing the cells in presence of increasing concentrations of ADR to obtain MCF7/ADR cells. The IC_50_ of MCF7/ADR cells were 5.05 fold for ADR and were higher compared with the parental MCF7 (Table 7). To examine the potential resistant mechanism, we performed a screening for resistant genes towards JNJ treatment, and identified that XIAP may plays a key role in JNJ-induced drug resistance in MCF7/ADR cells. XIAP is one of member in inhibitors of apoptosis (IAP) family, which includes c-IAP-1 and 2, Drosophila IAP-1 and 2 (DIAP-1 and 2), and BRUCE (BIR domain containing Ubiquitin Conjugating Enzyme) [24–26]. By knockdown XIAP by siRNAs, we were able to reduce the resistant effect in MCF7/ADR cells treated with JNJ.

**Table 7:** The primers used in the promoter/enhancer position and transcription factor AP-1 binding sites of human XIAP gene

regions	Gene	Sequence	the scope of the amplificated XIAP gene sequence
1	XIAP	5′- GTGGCTCACGCCTGTAATC-3′ 5′-TCGGTTCACTGCAAGTTCTG −3′	−1750 to −1450
2	XIAP	5′-GGCTCACGCCTGTAATCCTA-3′ 5′-ACTCCCGGGTTCCAGAGAT-3′	−1384 to −1121
3	XIAP	5′-GGAACCGAGAAGCTGACCTA-3 5′-GACAGTGCTTATCTTACAGGGTTG-3′	−340 to −1
4	XIAP	5′-TCACCAGCTAATTTCCTCTTCC-3′ 5′-CTGCAGGATTGCCTTCCTAA-3′	623 to 900
5	XIAP	5′-AGGTGGAGAGGGCTACTGCT-3′ 5′-AGGGTGGAGTGTGTCTGGAG-3′	800 to 1100
6	XIAP	5′-GGGTTTGACTAAGATCCCTTCC-3′ 5′-CCACCTTCGAAAAAGGAACA-3′	1050 to1350
7	XIAP	5′-GTAGAGGTGGGGTTTCACCA-3′ 5′-ACCATCTCAGCTCACTGCAA-3′	2608 to 2929

Further, we next to examine regulation of XIAP in MCF7/ADR cells treated with JNJ by analysis XIAP gene promoter. We identified that transcription factor AP-1 can recognize the promoter/enhancer regions in XIAP gene, TPA-responsive elements (TREs), 5′-TGAG/CTCA-3′, a conserved 9 bp motif shared by inducible genes [27] and a revised motif with AP-1 binding [28]. AP-1 is dimeric transcription factor of a ubiquitous family consisting of the Fos and Jun families of the DNA binding proteins [29, 30]. The activation of AP-1 was associated with the formation of c-Jun-c-Jun and c-Jun-c-Fos homodimers [31]. Jun proteins can form homodimer, while Fos protein can form heterodimer with Jun protein [32, 33]. Through formation of the complex, they can recognize different sequence elements in the promoters/enhancers of the target genes [34], and functioned in a various cellular processes such as promoting apoptosis or increasing cell survival under some certain physiological conditions [35]. In MCF7/ADR cells, JNJ treatment increased expression of c-Fos in both mRNA and protein level. c-Fos is frequently overexpressed in tumor cells and has oncogenic activity [36, 37]. Targeting c-Fos can abrogate intrinsic resistance to tyrosine-kinase inhibitor therapy in BCR-ABL-induced leukemia [38]. Previous studies have shown that c-Fos can support growth of peripheral and central nervous system tumors in humans and mice [39] and suppress differentiation-induced skin cancer [40]. Overexpression of c-Fos enhances the head and neck squamous cell carcinoma (HNSCC) cells’ promotion of tumor growth [41]. These reports suggest that activating c-Fos may induce cancer deterioration, and it is coincide with the result we identified in MCF7/ADR cells.

Previous study shows that via AP-1-dependent upregulation Notch-1 inhibition restores TRAIL-mediated apoptosis in MDA-MB-231 breast cancer cells [42], and LAD cells [43]. In our study, AP-1 was found to be recruited in the XIAP gene promoter and enhancer region at positions from −1750 to −1450 and from 2608 to 2929, and promote XIAP gene transcription after JNJ treatment. Further we validated the translocation of AP-1 in the nucleus and activation of AP-1 formation by increasing c-Fos expression level. The activation of c-Fos/AP-1/XIAP signaling pathway is confirmed in other cancer type such as liver cancer. However, JNJ treatment in naïve MCF7 cells, we observed the increasing sensitivity phenotype towards ADR treatment, which was consistent with growth arrest effects of JNJ reported in many cancer types such as multiple myeloma (MM) [44], lung cancer cells [14], rhabdomyosarcoma cells [17], synovial sarcoma [45], and cutaneous T-cell lymphoma (CTCL) [46]. However, treatment with JNJ did not rescue severe spinal muscular atrophy (SMA) mice with organ impairment [47]. Recent studies have shown that hampered AP-1 can induce apoptosis, such as downregulation of AP-1 expression inhibited MMP-2 expression in SCC9 cells *in vitro* [48], via inhibiting the Raf-1/AP-1 pathway reduced P-glycoprotein (P-gp) expression [49]. The rapid and sustained downregulation of c-Fos and its homodimers AP-1 nuclear translocation inhibited by JNJ may contribute to the drug sensitive effects of XIAP in MCF7 cells.

## MATERIALS AND METHODS

### Chemicals and reagents

The 3(4, 5dimethylthiazol2yl)2,5diphenyltetrazolium bromide (MTT) were bought from Sigma-Aldrich (St Louis, MO). Primary antibodies against c-Fos, AP-1, XIAP were bought from ImmunoWay Biotechnology Company in USA. A primary antibody against c-Jun was bought from Abcam. PrimeScript RT reagent Kit and SYBR Premix Ex Taq TM were purchased from TaKaRa.E.Z.N.AR HP Total RNA Kit, the product of Omega Bio-Tek (Doraville, USA) [50]. Smart pool siRNA against human XIAP (si-XIAP) and control (siNC), Smart pool siRNA against human HDAC_2_ (si-HDAC_2_) and control (siNC) were gained from RiboBio (Guangzhou, China). Adriamycin (ADR) was purchased from Zhejiang HISUN Pharmaceuticals Co. (Zhejiang, China) [50]. CHIP assay kit was produced from Millipore. Goat anti-Rabbit IgG-FITC was produced from Bioworld Technology, Inc.

### Cell culture

MCF7 (Adriamycin-sensitive) cell lines were purchased from the Culture Collection of the Chinese Academy of Sciences (Shanghai, China), and MCF7/ADR (Adriamycin-resistant) were cultured by our lab. We use 10% fetal bovine serum and RPMI 1640medium (Gibco BRL)under a humidified 5% CO_2_ atmosphere 37°C cultivate cells.

### MTT assay

The 3(4, 5dimethylthiazol2yl)2,5diphenyltetrazolium bromide(MTT; SigmaAldrich, St. Louis, MO, USA) assay was used to determine drug sensitivity. MCF7 and MCF7/ADR cells were seeded into 96well plates at a concentration of 5 × 10^3^ cells/200 μl/well. Cells were incubated at 37°C in a humidified 5% CO_2_ incubator. Following 48 h treatment with specific concentrations of the anticancer drugs ADR, JNJ (50 nM; Purchased from Selleck.) plates were added to standard tissue incubator conditions. Medium was removed and cells were solubilized in 150 μl DMSO. The intensity of formazan was measured at 490 nm using an automated microplate spectrophotometer (iMark; BioRad, Hercules, CA, USA). The survival rate was calculated as (OD value of the treated group/OD value of untreated group) x 100%. We performed three times in every experiment.

### Analysis of drug resistance reversal activity

Viability of MCF7 and MCF7/ADR cells following treatment with ADR in the presence or absence of JNJ (50 nM) was analyzed by MTT assay. Following plotting of the doseresponse curve, the IC_50_, the concentration of drug inhibiting 50% of cells, was calculated, from which reversal fold was calculated. siRNA inhibition of XIAP HDAC_1_ and HDAC_2_ expression. A 96-well plate was used to cultivate cells for 24 h and 5 × 10^3^ cells/well were cultured. They were then transfected with 100 pmol siRNA mixed with Lipofectamine 2000 reagent in serum reduced medium according to the manufacturer's instructions. Medium was changed to complete culture medium 4 h later, and the cells were incubated at 37°C in a CO_2_ incubator for another 24 h before harvest.

### Quantitative real-time PCR

After dealing as indicated, total mRNA of cells was extracted with TRIZOL reagent. First strand of cDNA synthesis was generated from 2 μg total RNA using oligo-dT primer and Superscript II Reverse Transcriptase (GIBCO BRL, Grand Island, NY, USA). Quantitative Real-Time PCR was carried on an iCycler (Bio-rad, Hercules, USA) using confirmed primers and SYBR Premix Ex Taq II (Takara, Japan) for detection. The cycle number when the fluorescence first reached a preset threshold (Ct) was used to quantify the initial concentration of individual templates for expression of mRNA of genes of interest. Primer pairs were as follows: c-Fos, forward 5′-GAATCCGAAGGGAAAGGAATAAG-3′ and reverse 5′-CTTCTAGTTGGTCTGTCTCCGCTT-3′; c-Jun, forward 5′-AGCATGACCCTGAACCTGG −3′ and reverse 5′-CCGTTGCTGGACTGGATT −3′; XIAP, forward 5′-GAAAGGTGACTGGGAAAGCA −3′ and reverse 5′-GAGCAGGGTGGAGTGTGTCT −3′; GAPDH, forward 5′- GCA CCGTCAAGGCTGAGAAC −3′ and reverse 5′-TGGTGA AGACGCCAGTGGA −3′.

### Western blotting analysis

The cells were washed three times with ice-cold phosphate buffer solution (PBS) and then lysed in lysis buffer containing 50 mM Tris-HCl (pH7.6), 150mM NaCl, 1mM EDTA, 1% NP-40, 0.5% Nadeoxycholate, 5 mg/mL aprotinin, 5 mg/mL leupeptin, and 1 mM phenylmethylsulfonyl fluoride. Lysates were cleared by centrifugation and denatured by boiling in Laemmli buffer. Equal amounts of protein samples were loaded per well and separated on SDS-polyacrylamide gels and then electrophoretically transferred onto PVDF membranes. Following blocking with 5% nonfat milk at room temperature for 2 h, membranes were incubated with primary antibodies (1:1000 dilution) at 4°C overnight and then incubated with HRP-conjugated secondary antibodies (1:5000 dilution) for 2 h at room temperature. Specific immune complexes were detected using Western Blotting Plus Chemiluminescence Reagent (Life Science).

### Flow cytometry analysis

To the detection of apoptosis, MCF7/ADR cells were directly stained with PI and Annexin V. Subsequently, using PBS to wash and resuspend cells. The fluorescence data were got using a flow cytometry machine (EXLTM, Beckman Coulter), and the data were analyzed using FlowJo software.

### Chromatin immunoprecipitation (ChIP)

DNA is sheared to the appropriate length with 3–4 sets of 10-second pulses using a Cole Parmer, High intensity Ultrasonic Processor/Sonicator, 50-watt model equipped with a 2 mm tip and set to 30% of maximum power. Stimulate or treat 1 × 10^6^ cells on a 10 cm dish as appropriate. Cross link AP-1 to DNA by adding formaldehyde directly to culture medium to a final concentration of 1% and incubate for 10 minutes at 37°C. Aspirate medium wash cells twice using ice cold PBS containing protease inhibitors Scrape cells into conical tube. Pellet cells for 4 minutes at 2000 × g at 4°C. Use SDS Lysis Buffer to dissolve precipitated SDS and add protease inhibitors. Resuspend cell pellet in SDS Lysis Buffer and incubate on ice. Sonicate lysate to shear DNA to lengths between 200 and 1000 base pairs. Dilute the sonicated cell supernatant 10fold in ChIP Dilution Buffer, adding protease inhibitors as above. This is done by adding 1800 μL ChIP Dilution Buffer to the 200 μL sonicated cell supernatant for a final volume of 2 mL in each PCR condition. A portion of the diluted cell supernatant 1% (~20 μL) can be kept to quantitate the amount of DNA present in different samples at the PCR protocol. This sample is considered to be input/starting material material and needs to have the AP-1-DNA crosslinks reversed by heating at 65°C for 4 hours. To reduce nonspecific background, pre-clear the 2 mL diluted cell. By brief centrifugation pellet agarose and collect the supernatant fraction. The immunoprecipitating antibody added to the 2 mL supernatant fraction was rotation and incubate overnight at 4°C.a no-antibody immunoprecipitation was performed by incubating the supernatant fraction with 60 μL of Salmon Sperm DNA/Protein A Agarose-50% for a negative control and slurry for one hour at 4°C with rotation and collect the antibody/AP-1complex. Tender centrifugation Pellet agarose (700 to 1000 rpm at 4°C, ~1min). Remove the unbound supernatant and non-specific DNA. The protein Aagarose/antibody/AP-1 complex was washed for 3–5 minutes on a rotating platform with 1ml of each of the buffers. The sample is now a protein A/antibody/AP-1/DNA complex, and we can use it for PCR assay.

### PCR protocol to amplify DNA that is bound to the immunoprecipitatedAP-1

Prepare Fresh elution buffer. Adding 250 μL elution buffer to the pelleted protein Aagarose/antibody/AP-1 complex to elute the AP-1 complex from the antibody. Vortex and incubate 15 minutes at room temperature with rotation. Roll down agarose, and transfer the supernatant fraction to other tube and do again. Merge eluates (total volume = ~500 μL). 20 μL 5 M NaCl was added to the merged eluates (500 μL) and overturn AP-1-DNA crosslinks in heating at 65°C condition for 4 hours. At this step the sample can be stored and −20°C and the protocol continued the next day. Add 10 μL of 0.5 M EDTA, 20 μL 1 M Tris-HCl, pH 6.5 and 2 μL of 10 mg/mL Proteinase K to the combined eluates and incubate for one hour at 45°C. With phenol/chloroform extraction and ethanol precipitation DNA was recovered. An inert carrier, 20 μg glycogen, can make DNA pellet visualize. Use 70% ethanol to wash pellets and air dry. Use an appropriate buffer for PCR to resuspend pellets.

### qPCR of ChIP enriched DNA

Immunoprecipitated DNA fragments were analysed by real-time PCR. Primers used are listed in Table 3. All samples were amplified using a set of biological replicates with three technical replicates used per sample.

### Immunofluorescence assay

MCF7 cells were incubated with JNJ (50 nM). After 48 h, cells were washed with PBS and fixed with 4% paraformaldehyde for 30 min at room temperature, after that, cells were washed with PBS and permeabilized in 0.3% Triton X-100 (prepared in PBS) for 30 min at 37°C. Then normal serum working liquid goat closed at room temperature incubated 30 min. Then cells were added antibody against AP-1 and incubated overnight. Then washed with PBS and added goat anti-rabbit IgG-FITC incubated 45 min. Then cellular nuclei were counterstained with 5 μg/ml 4,6-diamidino-2-phenylindole (DAPI). Similarly, MCF7/ADR cells were incubed with JNJ (50 nM). After washing and fixation, the cells were incubated with antibody against AP-1 (dilution 1:100) for 16h at room temperature and then with goat anti-rabbit IgG-FITC for another 45 min at room temperature. Cellular nuclei were counter stained with DAPI, and images were made with a confocal microscope (LSM710, ZeissGermany).

### Statistical analysis

Data were represented as mean ± SD in triplicate, and each experiment was repeated twice or thrice. Student's *t*-test was employed to analyze the data and a two-side *p* value less than 0.05 was considered statistically significant.

## CONCLUSIONS

Our study identified a new regulatory pathway, c-Fos/AP-1/XIAP, that contributes the induction of drug resistant in breast and liver cancer cells. JNJ is able to up-regulate c-Fos expression in both mRNA and protein levels in MCF7/ADR and HepG2 cells. The molecular mechanism underlying resistance induced by JNJ is induction of c-Fos and activation of AP-1 formation, thus increasing expression of XIAP, an anti-apoptosis factor, which causing resistance towards serious ADR treatment. Our study identified a potential therapeutic target in those resistant cancer cells and concluded that JNJ is not an available adjunct treatment for those patients with chemo-resistance.

## References

[1] Butler EB, Zhao Y, Munoz-Pinedo C, Lu J, Tan M (2013). Stalling the engine of resistance: targeting cancer metabolism to overcome therapeutic resistance. Cancer Res.

[2] Luqmani YA (2005). Mechanisms of drug resistance in cancer chemotherapy. Med Princ Pract.

[3] Li J, Xu LZ, He KL, Guo WJ, Zheng YH, Xia P, Chen Y (2001). Reversal effects of nomegestrol acetate on multidrug resistance in adriamycin-resistant MCF7 breast cancer cell line. Breast Cancer Res.

[4] Livney YD, Assaraf YG (2013). Rationally designed nanovehicles to overcome cancer chemoresistance. Adv Drug Deliv Rev.

[5] Jabr-Milane LS, van Vlerken LE, Yadav S, Amiji MM (2008). Multi-functional nanocarriers to overcome tumor drug resistance. Cancer Treat Rev.

[6] Szakacs G, Paterson JK, Ludwig JA, Booth-Genthe C, Gottesman MM (2006). Targeting multidrug resistance in cancer. Nat Rev Drug Discov.

[7] Gottesman MM (2002). Mechanisms of cancer drug resistance. Annu Rev Med.

[8] Bolden JE, Peart MJ, Johnstone RW (2006). Anticancer activities of histone deacetylase inhibitors. Nat Rev Drug Discov.

[9] Marks PA, Jiang X (2005). Histone deacetylase inhibitors in programmed cell death and cancer therapy. Cell Cycle.

[10] Xargay-Torrent S, Lopez-Guerra M, Saborit-Villarroya I, Rosich L, Campo E, Roue G, Colomer D (2011). Vorinostat-induced apoptosis in mantle cell lymphoma is mediated by acetylation of proapoptotic BH3-only gene promoters. Clin Cancer Res.

[11] Yazbeck VY, Grant S (2015). Romidepsin for the treatment of non-Hodgkin's lymphoma. Expert Opin Investig Drugs.

[12] Heinicke U, Kupka J, Fichter I, Fulda S (2016). Critical role of mitochondria-mediated apoptosis for JNJ-26481585-induced antitumor activity in rhabdomyosarcoma. Oncogene.

[13] Arts J, King P, Marien A, Floren W, Belien A, Janssen L, Pilatte I, Roux B, Decrane L, Gilissen R, Hickson I, Vreys V, Cox E (2009). JNJ-26481585, a novel "second-generation" oral histone deacetylase inhibitor, shows broad-spectrum preclinical antitumoral activity. Clin Cancer Res.

[14] Bao L, Diao H, Dong N, Su X, Wang B, Mo Q, Yu H, Wang X, Chen C (2016). Histone deacetylase inhibitor induces cell apoptosis and cycle arrest in lung cancer cells via mitochondrial injury and p53 up-acetylation. Cell Biol Toxicol.

[15] Grant S, Dai Y (2012). Histone deacetylase inhibitors and rational combination therapies. Adv Cancer Res.

[16] Xue K, Gu JJ, Zhang Q, Mavis C, Hernandez-Ilizaliturri FJ, Czuczman MS, Guo Y (2016). Vorinostat, a histone deacetylase (HDAC) inhibitor, promotes cell cycle arrest and re-sensitizes rituximab- and chemo-resistant lymphoma cells to chemotherapy agents. J Cancer Res Clin Oncol.

[17] Heinicke U, Kupka J, Fulda S (2015). JNJ-26481585 primes rhabdomyosarcoma cells for chemotherapeutics by engaging the mitochondrial pathway of apoptosis. Oncotarget.

[18] Minotti G, Menna P, Salvatorelli E, Cairo G, Gianni L (2004). Anthracyclines: molecular advances and pharmacologic developments in antitumor activity and cardiotoxicity. Pharmacol Rev.

[19] Grossman HB, Natale RB, Tangen CM, Speights VO, Vogelzang NJ, Trump DL, deVere White RW, Sarosdy MF, Wood DP, Raghavan D, Crawford ED (2003). Neoadjuvant chemotherapy plus cystectomy compared with cystectomy alone for locally advanced bladder cancer. N Engl J Med.

[20] McClung EC, Wenham RM (2016). Profile of bevacizumab in the treatment of platinum-resistant ovarian cancer: current perspectives. Int J Womens Health.

[21] Kumar V, Palazzolo S, Bayda S, Corona G, Toffoli G, Rizzolio F (2016). DNA Nanotechnology for Cancer Therapy. Theranostics.

[22] Barrand MA, Heppell-Parton AC, Wright KA, Rabbitts PH, Twentyman PR (1994). A 190-kilodalton protein overexpressed in non-P-glycoprotein-containing multidrug-resistant cells and its relationship to the MRP gene. J Natl Cancer Inst.

[23] Mehta K (1994). High levels of transglutaminase expression in doxorubicin-resistant human breast carcinoma cells. Int J Cancer.

[24] Vasudevan D, Ryoo HD (2015). Regulation of Cell Death by IAPs and Their Antagonists. Curr Top Dev Biol.

[25] Birnbaum MJ, Clem RJ, Miller LK (1994). An apoptosis-inhibiting gene from a nuclear polyhedrosis virus encoding a polypeptide with Cys/His sequence motifs. J Virol.

[26] Crook NE, Clem RJ, Miller LK (1993). An apoptosis-inhibiting baculovirus gene with a zinc finger-like motif. J Virol.

[27] Angel P, Imagawa M, Chiu R, Stein B, Imbra RJ, Rahmsdorf HJ, Jonat C, Herrlich P, Karin M (1987). Phorbol ester-inducible genes contain a common cis element recognized by a TPA-modulated trans-acting factor. Cell.

[28] Iyer VR, Eisen MB, Ross DT, Schuler G, Moore T, Lee JC, Trent JM, Staudt LM, Hudson J, Boguski MS, Lashkari D, Shalon D, Botstein D (1999). The transcriptional program in the response of human fibroblasts to serum. Science.

[29] Hartl M, Vogt PK (1992). Oncogenic transformation by Jun: role of transactivation and homodimerization. Cell Growth Differ.

[30] Kappelmann M, Bosserhoff A, Kuphal S (2014). AP-1/c-Jun transcription factors: regulation and function in malignant melanoma. Eur J Cell Biol.

[31] Herdegen T, Skene P, Bahr M (1997). The c-Jun transcription factor--bipotential mediator of neuronal death, survival and regeneration. Trends Neurosci.

[32] Abate C, Curran T (1990). Encounters with Fos and Jun on the road to AP-1. Semin Cancer Biol.

[33] Curran T, Morgan JI (1987). Memories of fos. Bioessays.

[34] Curran T, Franza BR (1988). Fos and Jun: the AP-1 connection. Cell.

[35] Yin Y, Wang S, Sun Y, Matt Y, Colburn NH, Shu Y, Han X (2009). JNK/AP-1 pathway is involved in tumor necrosis factor-alpha induced expression of vascular endothelial growth factor in MCF7 cells. Biomed Pharmacother.

[36] Milde-Langosch K (2005). The Fos family of transcription factors and their role in tumourigenesis. Eur J Cancer.

[37] Durchdewald M, Angel P, Hess J (2009). The transcription factor Fos: a Janus-type regulator in health and disease. Histol Histopathol.

[38] Kesarwani M, Kincaid Z, Gomaa A, Huber E, Rohrabaugh S, Siddiqui Z, Bouso MF, Latif T, Xu M, Komurov K, Mulloy JC, Cancelas JA, Grimes HL (2017). Targeting c-FOS and DUSP1 abrogates intrinsic resistance to tyrosine-kinase inhibitor therapy in BCR-ABL-induced leukemia. Nat Med.

[39] Silvestre DC, Gil GA, Tomasini N, Bussolino DF, Caputto BL (2010). Growth of peripheral and central nervous system tumors is supported by cytoplasmic c-Fos in humans and mice. PLoS One.

[40] Guinea-Viniegra J, Zenz R, Scheuch H, Jimenez M, Bakiri L, Petzelbauer P, Wagner EF (2012). Differentiation-induced skin cancer suppression by FOS, p53, and TACE/ADAM17. J Clin Invest.

[41] Muhammad N, Bhattacharya S, Steele R, Phillips N, Ray RB (2016). Involvement of c-Fos in the Promotion of Cancer Stem-like Cell Properties in Head and Neck Squamous Cell Carcinoma. Clin Cancer Res.

[42] Portanova P, Notaro A, Pellerito O, Sabella S, Giuliano M, Calvaruso G (2013). Notch inhibition restores TRAIL-mediated apoptosis via AP1-dependent upregulation of DR4 and DR5 TRAIL receptors in MDA-MB-231 breast cancer cells. Int J Oncol.

[43] Huang J, Chen Y, Li J, Zhang K, Chen J, Chen D, Feng B, Song H, Feng J, Wang R, Chen L (2016). Notch-1 Confers Chemoresistance in Lung Adenocarcinoma to Taxanes through AP-1/microRNA-451 Mediated Regulation of MDR-1. Mol Ther Nucleic Acids.

[44] Deleu S, Lemaire M, Arts J, Menu E, Van Valckenborgh E, King P, Vande Broek I, De Raeve H, Van Camp B, Croucher P, Vanderkerken K (2009). The effects of JNJ-26481585, a novel hydroxamate-based histone deacetylase inhibitor, on the development of multiple myeloma in the 5T2MM and 5T33MM murine models. Leukemia.

[45] Laporte AN, Barrott JJ, Yao RJ, Poulin NM, Brodin BA, Jones KB, Underhill TM, Nielsen TO (2017). HDAC and Proteasome Inhibitors Synergize to Activate Pro-Apoptotic Factors in Synovial Sarcoma. PLoS One.

[46] Child F, Ortiz-Romero PL, Alvarez R, Bagot M, Stadler R, Weichenthal M, Alves R, Quaglino P, Beylot-Barry M, Cowan R, Geskin LJ, Perez-Ferriols A, Hellemans P (2016). Phase II multicentre trial of oral quisinostat, a histone deacetylase inhibitor, in patients with previously treated stage IB-IVA mycosis fungoides/Sezary syndrome. Br J Dermatol.

[47] Schreml J, Riessland M, Paterno M, Garbes L, Rossbach K, Ackermann B, Kramer J, Somers E, Parson SH, Heller R, Berkessel A, Sterner-Kock A, Wirth B (2013). Severe SMA mice show organ impairment that cannot be rescued by therapy with the HDACi JNJ-26481585. Eur J Hum Genet.

[48] Hsieh MJ, Chen JC, Yang WE, Chien SY, Chen MK, Lo YS, Hsi YT, Chuang YC, Lin CC, Yang SF (2017). Dehydroandrographolide inhibits oral cancer cell migration and invasion through NF-kappaB-, AP-1-, and SP-1-modulated matrix metalloproteinase-2 inhibition. Biochem Pharmacol.

[49] Wang F, Lv P, Gu Y, Li L, Ge X, Guo G (2017). Galectin-1 knockdown improves drug sensitivity of breast cancer by reducing P-glycoprotein expression through inhibiting the Raf-1/AP-1 signaling pathway. Oncotarget.

[50] Lu LL, Chen XH, Zhang G, Liu ZC, Wu N, Wang H, Qi YF, Wang HS, Cai SH, Du J (2016). CCL21 Facilitates Chemoresistance and Cancer Stem Cell-Like Properties of Colorectal Cancer Cells through AKT/GSK-3beta/Snail Signals. Oxid Med Cell Longev.

